# Characterizing Palestinian snake melon (*Cucumis melo* var. *flexuosus*) germplasm diversity and structure using SNP and DArTseq markers

**DOI:** 10.1186/s12870-018-1475-2

**Published:** 2018-10-19

**Authors:** Salam Y. Abu Zaitoun, Rana M. Jamous, Munqez J. Shtaya, Omar B. Mallah, Imad S. Eid, Mohammed S. Ali-Shtayeh

**Affiliations:** 1 Biodiversity and Environmental Research Center, BERC, Til, Nablus, Palestine; 2 Department of Plant Production and Protection, Faculty of Agriculture, An-Najah University, Tulkarm, Palestine

**Keywords:** Palestinian snake melon landraces, Genetic diversity, *Cucumis melo* var. *flexuosus*, Population structure, SNP, Genotyping by sequencing, DArT-seq

## Abstract

**Background:**

Crop landraces embody a source of beneficial genes potentially providing endurance to environmental stress and other agronomic qualities including yield. Our study included 88 snake melon accessions (*Cucumis melo* var. *flexuosus*) collected from 9 districts in the Palestinian West-Bank. These accessions represent four landraces of Palestinian snake melon: Green, and White Baladi, and Green, and White Sahouri.

**Results:**

This is the first report on successful application of genotyping by sequencing in snake melon. Nine thousand seven hundred fifty single-nucleotide polymorphism (SNP) and 7400 DArTseq genetic markers were employed to evaluate genetic biodiversity and population structure of Palestinian snake melon germplasm collection. Clustering based on neighbor-joining-analysis, principle coordinate and Bayesian model implemented in Structure showed that patterns of genetic diversity of snake melon landraces depends on their geographical source and unraveled the presence of two major local landraces (*Sahouri*, and *Baladi*) with accessions from each group clustering together. A significant correlation was observed between both types of markers in Mantel correlation test. A significant association between genetic and geographic matrices (*P* < 0.0001) was also detected. AMOVA indicated that majority of variation (90%) was due to the difference within accessions.

**Conclusion:**

The Palestinian landraces seem to have unique genes that may allow the enhancement of the global snake melon gene pool and developments of the plant production worldwide. Our subsequent objective is to detect genotypes with promising qualities and to conduct association mapping studies concentrating on Fusarium-wilt resistance, yield, and environmental stresses.

**Electronic supplementary material:**

The online version of this article (10.1186/s12870-018-1475-2) contains supplementary material, which is available to authorized users.

## Background

Melon, *Cucumis melo* L. (family Cucurbitaceae), is a morphologically diverse, out-crossing horticultural crop of broad economic importance. *Cucumis melo* is considered as a polymorphous species, showing wildly diverse fruit morphology [1, 2]. The species comprises two subspecies, *C. melo L. subsp*. *agrestis* and *C. melo L. subsp*. *melo*, differentiated by the pubescence on the female hypanthium [3]. It encompasses 15 different groups or varieties of which 5 varieties are belonging to the ssp. *agrestis*, and 10 varieties including *flexuosus* are belonging to the ssp. *melo* [4].

The snake melon (*Cucumis melo subsp. melo* L. var. *flexuosus* (L.) is an ancient crop in many parts of the world including the Middle East and Middle Asia [5]. Most melon fruits have a ratio of 1:1 of length to width, while, the flexuosus group cultivars are characterized by their fruits with a ratio of length to broadest width of 4:1 or higher [6, 7]. Snake melon is a traditionally cultivated vegetable crop grown in summer in tropical and subtropical conditions. The fruits are usually harvested while they are still green and consumed cooked, pickled or as fresh vegetables in some Asian and African countries [8].

Crop landraces represent a source of useful genes with a high capacity to endure biotic and abiotic stresses, resulting in high yield stability, disease resistance, and moderate yield level under a low input agricultural system [9]. Farmers in Palestine have conserved the diversity of snake melon, in the form of four landraces, as a primary gene pool: White Baladi (WB), Arabic Baladi Abiadh; Green Baladi (GB), Baladi Akhder; White Sahouri (WS), Sahouri Abiadh; and Green Sahouri (GS), Sahouri Akhder [10]. However, melon landraces are currently threatened by severe genetic erosion caused by improper management and the replacement of local cultivars by improved ones.

Palestinian snake melon landraces show large variation in fruit attributes such as size, shape, color, taste, texture, and biochemical composition [10]. Such variation provides breeders with a rich genetic resource; which necessitates the need to understand their genetic diversity.

An understanding of the genetic diversity of snake melon is important to prevent the deterioration of cultivated gene pools and to guide the use of genetic resources in breeding programs [10, 11]. Several molecular markers have been successfully used to investigate the phylogenetic relationships and the genetic diversity of melons and its close relatives. Restriction fragment length polymorphisms (RFLPs), random amplified polymorphic DNA (RAPD), simple sequence repeat (SSR), inter-simple sequence repeat (ISSR), and amplified fragment length polymorphism (AFLP) [12–15] have been used. Most of these marker systems are restricted in their applications because of the limitation on their availability, expensive when analyses are conducted on a large scale, and time-consuming process, which made them eventually outdated [16].

Development of molecular markers has been primarily motivated by the throughput, level of reproducibility, and the cost of the detection method. Among various types of molecular markers, single-nucleotide polymorphisms (SNPs) are the most ample in a genome and suitable for analysis on a wide range of genomic scales as they are present in a large number and combine excellent features such as wide genomic distribution, and high reproducibility. SNP markers are commonly used to describe genetic diversity, and they have many advantages related to their ease of data management along with their elasticity, speed, and low cost [17–20].

The Diversity Arrays Technology (DArT) have been developed more than 15 years ago to meet the resolution and speed essential for a variety of applications in genomics and molecular breeding of plants which generate more polymorphic markers and comprehensively cover the genome [21, 22]. DArT has gained a growing interest globally as it has conveniently satisfied the requirements of genome coverage, high throughput and transferability for a large number of diverse plant species [23, 24]. DArT has proven to be useful for the analysis of different crops including rice, *Oryza sativa* L. [21], eucalyptus, *Eucalyptus grandis* Hill ex Maiden [25], barley, *Hordeum vulgare* L. [26], wheat, *Triticum aestivum* L. [27, 28], and cassava, *Manihot esculenta* Crantz [29].

More recently with the advent of new sequencing technologies (so-called Next Generation Sequencing or NGS), genome complexity reduction for genotyping has been taken to a higher level with different complexity reduction methods, similar to the one used in DArTseq (Sequencing-Based Diversity Array Technology) [30]. This method depends on genome complexity reduction by using restriction enzymes, followed by hybridization to microarrays to concurrently assay hundreds to thousands of markers across a genome.

DArTseq technology is a united one-step procedure of SNP discovery and genotyping; it enables a substantial discovery of SNPs in a wide variety of non-model organisms and provides measures of genetic divergence and diversity within the major genetic groups that comprise crop germplasm [31].

DArTseq and SNP markers based on GBS technology have been successfully used for genotyping, genetic diversity, population structure studies, and for genetic mapping [27, 32–38]. However, genetic diversity and population structure of *C. melo* ssp. *melo* var. *flexuosus* revealed by DArTseq and SNP markers together have not been reported yet.

Understanding the population structure of a group of landraces is important to unravel the relationships between phenotypes and genotypes [39]. Population structure analysis using DArTseq technique covers the entire genome and provides a complete picture of the population based on complexity reduction by the use of restriction enzymes targeting gene-rich regions and NGS. Today the GBS assay is considered one of the most appropriate approaches for concurrent large-scale SNP discovery and genotyping [40]. GBS is widely used to detect polymorphism in plant species with sequence information [41, 42].

Recently GBS approach has been successfully used to characterize patterns of genetic diversity and genomic features within *Cucumis melo* germplasm [43]. However, there are no reports of the application of GBS (DArTseq) in snake melon. Therefore, the objective of our present study was to explore the population structure and genetic diversity of Palestinian snake melon using GBS (DArTseq).

## Methods

### Plant materials

The plant material used in this study consists of 88 accessions representing the four Palestinian snake melon local landraces collected in the Palestinian West Bank and deposited in the Community Based Gene Bank of the Biodiversity & Environmental Research Center- BERC (See Additional file 1). These accessions were collected from different geographical areas of the West Bank. Ten mature fruits of the four Palestinian snake melon landraces: WB, GB, GS, and WS were collected from each of 50 snake melon fields (populations) distributed in 9 districts in the West Bank during the summer of 2014. Seeds (representing one accession) were extracted from each fruit. A total of 500 accessions were deposited in the BERC seed bank until used. The following summer of 2015, seeds representing 200 snake melon accessions were planted in an experimental field at BERC Agricultural Station, Til village, Nablus (32° 19’ N, 35° 20′ E and 2039 Ft above sea level), of these, leaf samples of 88 accessions representing the four Palestinian landraces: GB, 20; WB, 28; GS, 16, and WS, 24 were collected for genotyping-by-sequencing analysis.

### DNA extraction

The CTAB method, as recommended by the Diversity Arrays Technology Pty. Ltd., Yarralumla, Australia (http://www.diversityarrays.com/), was used to extract genomic DNA from the leaves of each accession. The quality and quantity of the obtained DNA were checked using Epoch Spectrophotometer (Biotek USA) and further checked on 0.8% agarose gel run in TAE buffer at 80 V. DNA concentrations of less than 50 ng μl^− 1^, and degraded DNA was eliminated.

### Genotyping by sequencing (GBS) analysis

Genotyping by sequencing (GBS) analysis of the 88 snake melon accessions was carried out by using a whole genome profiling service for DArTseq and SNP markers, Amplicons containing SNP were located on the melon genome [4]. 200 μl of 100 ng μl^− 1^ were sent to DArT Pty. Ltd., Yarralumla, Australia (http://www.diversityarrays.com/) for DArTseq and SNP analyses using GBS 1.0 V array protocol as described by Wenzl et al., [26], and Akbari et al., [27]. Raw sequence data of each clone is given in Additional file 2.

### Statistical analysis

DArTsoft v.7.4.7 (DArT P/L, Canberra, Australia) was used to analyze the images from DArTseq and SNP platforms. The markers (DArTseq and SNP) were scored using DArTsoft v.7.4.7 as binary data (1 present, 0 absent, for a marker in the genomic DNA of each accession) [27]. Several quality parameters for DArTseq and SNP markers including call rate, polymorphic information content (PIC), and reproducibility, were automatically computed by the DArT software.

The DARwin 6 software was used to calculate the genetic distance coefficients between collected snake melon accessions based on the proportions of shared alleles obtained from both DArTseq and SNP markers for each accession [44]. The software was also employed to construct the neighbor-joining trees based on pair-wise genetic distances among snake melon accessions.

The correlation matrices between DArTseq and SNP marker systems were determined using the Mantel test on XLSTAT software. Principal Coordinate Analysis (PCoA) based on the standardized covariance of calculated genetic distances for SNP and DArTseq markers and the analysis of molecular variance AMOVA were performed in GenAlex 6.41 software.

### Population structure analysis

The Bayesian Model-based clustering algorithm implemented in the STRUCTURE software 2.3.4 was employed to explore the genetic structure of Palestinian snake melon collection. To determine the number of hypothetical clusters (K), define populations and assign individual accessions to certain subpopulations based on genetic data, admixture and shared allele frequencies model was employed [45]. Numbers in the range from 1 to 10 were assumed for K. The initial burn-in period, for each run, was set to 10,000 with 100,000 MCMC (Markov chain Monte Carlo) iterations, with no previous information on the source of accessions. The most suitable value of K was calculated using the ΔK method as used in Structure Harvester web page [46].

## Results

### Markers quality and diversity

A total of 7400 DArTseq and 9750 SNP markers were generated for the 88 snake melon accessions representing the four local landraces (GB, WB, GS, and WS) from different geographical areas demonstrating the diversity present in the West Bank. The chromosomal locations of 4954 DArTseq markers and 8970 SNP markers have been provided by the Diversity Array Technology, Pvt., Ltd., Australia, on the melon genome. About 31% of the DArTseq markers and 8% of SNP markers have been reported for the first time in these accessions and their chromosomal locations are not known yet. The average polymorphism information content (PIC) of DArTseq and SNP markers were 0.192 and 0.234, respectively (Table 1). Call rate and reproducibility percentage were used to assess the quality of the markers. The average call rate of all markers that shows the fidelity of the final scores and produces the number of scored slides versus the maximum number of potential scores was 92.48% for DArTseq markers and 95.24% for SNP markers. The reproducibility of DArTseq and SNP markers used in the analyses were 99.4%, and 98.26%, respectively, showing high percent reproducibility, and consistent marker score.

**Table 1:** Genetic diversity of the four Palestinian snake melon landraces using the DArTseq and SNP data sets

	Number of markers	Average PIC	Average Call rate (%)	Reproducibility (%)	Landrace	Na	Ne	I	He	P (%)
DArTseq markers	7400	0.192	92.48	99.4	GB	2.14	1.49	0.45	0.27	77.2
					GS	1.98	1.45	0.40	0.24	68.5
					WB	2.17	1.48	0.44	0.26	77.0
					WS	2.04	1.42	0.39	0.23	69.3
SNP Markers	9750	0.234	95.24	98.2	GB	1.81	1.48	0.42	0.28	81.4
					GS	1.75	1.43	0.38	0.25	75.3
					WB	1.85	1.50	0.43	0.29	84.7
					WS	1.81	1.45	0.40	0.26	80.7

*Abbreviations*: *Na* Number of different alleles, *Ne* Number of effective alleles, *I* Shannon’s diversity index, *He* Expected heterozygosity, *P* Polymorphic loci

The Shannon’s diversity index (I) was employed in this study to quantify the genetic diversity of the four snake melon landraces using the DArTseq data set. The Shannon’s diversity index (I) was 0.45 for GB, 0.40 for GS, 0.44 for WB and 0.39 for WS (Table 1). The number of effective alleles (Ne) was 1.49, 1.45, 1.48 and 1.42 for each landrace, respectively, while the expected heterozygosity (He) was 0.27, 0.24, 0.26, and 0.23, for these landraces with an average of 0.25. The percentage of polymorphic loci (P%) ranged between 68.5–77.2 with an average of 73.0.

Using the SNP data set, Shannon’s diversity index (I) ranged between 0.38 for GS and 0.43 for WB (Table 1). The number of effective alleles (Ne) was 1.48 for GB, 1.43 for GS, 1.50 for WB and 1.45 for WS, while the expected heterozygosity (He) was 0.28, 0.25, .0.29, and 0.26, for these landraces with an average of 0.27. The percentage of polymorphic loci (P%) ranged between 75.3–84.7 with an average of 80.5.

### Population structure of the Palestinian snake melon germplasm

**Fig. 1:**
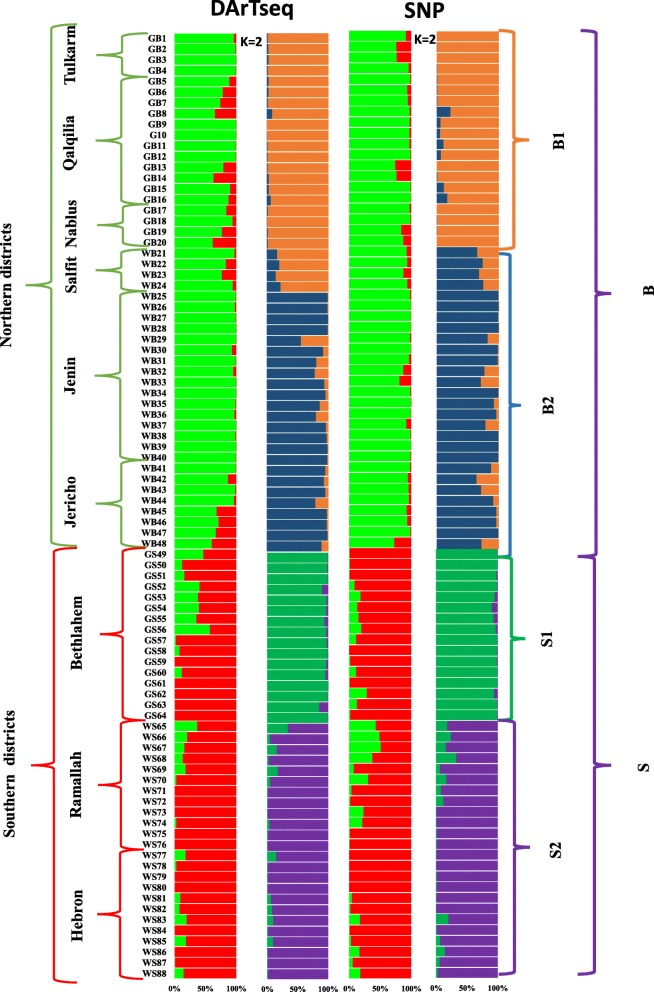
Population STRUCTURE analysis of 88 snake melon accessions using 7400 DArTseq and 9750 SNP markers. The proportion of membership in each group and subgroups at ΔK = 2 as defined with a model-based clustering method from Pritchard et al. [45] based on DArTseq and SNP markers. The collection sites and local landrace of each group and subgroup were analyzed and found well align which mentioned next to each

Population genetic structure of the Palestinian snake melon was explored by implementing an admixture-based clustering model in the software STRUCTURE. The suitable value of K was estimated using Evanno method, a model with two subpopulations (K = 2) was the most suitable, indicating the presence of two main groups Baladi (B), and Sahouri (S) in the total collection (Fig. 1). Based on the membership probabilities of ≥0.75 (See Additional file 3), most of the accessions (55%) were assigned to group B, whereas the remaining accessions (45%) were assigned to group S. The B comprised of 48 accessions, in this group, 36 accessions were from northern districts of the West Bank, while 12 accessions from the middle area of the West Bank. The S group comprised of 40 accessions, of which 12 accessions were from the middle area of the West Bank, and the remaining 28 accessions were from the southern districts of the West Bank (Table 2). Each of the two main groups B and S was further subdivided into two subgroups (B1 and B2 in B, and S1 and S2 in S), which were inferred from the ΔK values that gave K = 2 for both B and S (Fig. 1).

**Table 2:** Geographic origin and average membership probability of the snake melon germplasm assigned by the software STRUCTURE to groups Baladi (B), and Sahouri (S) and subgroups (B1, B2, S1, S2)

District	Landrace	No. of accessions (%)	Group/subgroup	Average Membership Probability to Group/Subgroup	
				DArTseq Markers	SNP Markers
Nablus	Green baladi	4 (4.5)	B/B1	0.79/0.98	0.92/0.99
Qalqilia	Green baladi	12 (13.5)	B/B1	0.86/0.97	0.94/0.93
Tulkarm	Green baladi	4 (4.5)	B/B1	0.99/0.98	0.85/0.99
Jericho	White baladi	8 (9)	B/B2	0.81/0.93	0.93/0.85
Jenin	White baladi	16 (18)	B/B2	0.99/0.90	0.97/0.93
Salfit	White baladi	4 (4.5)	B/B2	0.88/0.81	0.92/0.71
Bethlehem	Green sahouri	16 (18)	S/S1	0.80/0.97	0.91/0.98
Hebron	White sahouri	12 (13.5)	S/S2	0.92/0.96	0.94/0.95
Ramallah	White sahouri	12 (13.5)	S/S2	0 .93/0.93	0.77/0.89

The geographic origin of the snake melon germplasm assigned by the software STRUCTURE to groups (B and S) and subgroups (B1, B2, S1, S2) and their membership probability using both markers systems was presented in Table 2. The geographical distribution of the accessions in the two groups covered different sampling districts in the West Bank. Bayesian assignation to groups, based on maximum membership probability for each individual, was largely consistent with the folk landraces classification or site of collection. All accessions were assigned to the corresponding group (Table 2, Fig. 1).

B1 was a typical GB landrace and included 20 accessions collected from 5 different locations in the northern districts of the West Bank (Qalqilia, Tulkarm, and Nablus). B2 contained the WB landrace, with 12 (13.5%) accessions were from the middle area of the West Bank (Salfit and Jericho) and 16 accessions from Jenin in the northern area of the West Bank. The mean proportions of membership for WB and GB in cluster B were 0.92 and 0.87 in DArTseq data set and 0.93 and 0.95 in SNP data set.

S1 comprised GS accessions collected from four different locations in Bethlehem district, and S2 was the 24 WS accessions collected from six different locations in Ramallah and Hebron districts. The mean proportion of membership of GS and WS cultivars in cluster S were 0.91 and 0.80 in DArTseq dataset and 0.94 and 0.86 in SNP data set.

**Fig. 2:**
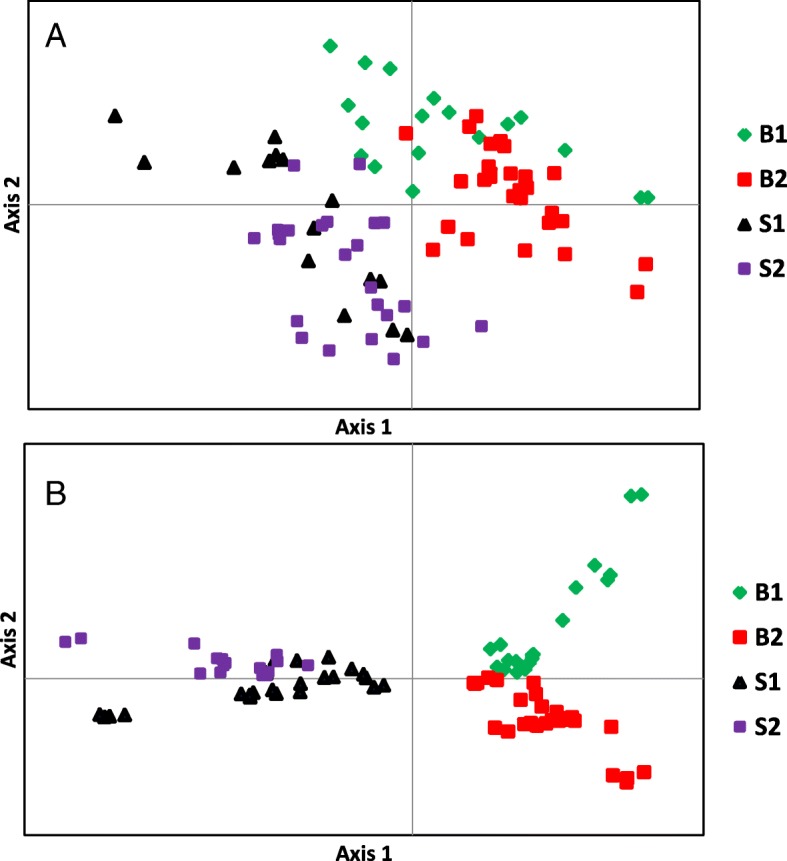
Principal coordinate analysis of snake melon accessions based on (**a**) DArTseq and (**b**) SNP markers

Bayesian assignation to groups was largely consistent with the landraces local classification. PCoA was performed to measure the variation in the snake melon whole collection in terms of accession grouping and pattern of variation. The first five principal coordinates explained 19.7% of the total variations in DArTseq and 23% in SNP markers. Using the first two axes, PCoA produced results similar to the STRUCTURE analysis, four clusters labeled with different symbols and colors, which conformed to the four subgroups in the STRUCTURE analysis, were identified though some accessions distributed far from the clusters (Fig. 2). It is worth noting that for the SNP data, the accessions clustered together more tightly and groups have less overlap than the DArT markers. This might be attributed to the fact that SNP are abundant in plant genomes, and SNP markers explained a higher percentage of the total variation as mentioned above.

### Diversity of Palestinian snake melon germplasm

The Nei genetic distance index (GD) values were calculated among the 88 snake melon accessions using the DArTseq markers data set. The average GD among all accessions was 0.239. One indistinguishable pair of accessions (GS63/GS64) both were collected from Bethlehem, with GD = 0, whereas the highest GD (0.356) was detected between accession GB1 from Nablus and (GB9 and GB10) from Qalqilia.

Following the estimation of diversity among all accessions, the average genetic distances among the pairs of accessions within each group were calculated. Both groups (*Baladi* and *Sahouri*) were found to possess a comparable level of genetic diversity. On the other hand, no clear differentiation was detected among the *Sahouri* group accessions. The average genetic distance between *Sahouri* and *Baladi* accessions was 0.25. The genetic distances among pairs of accessions within each group ranged from 0.06 to 0.36 (av.0.24) in *Baladi*, whereas it varied from 0.03 to 0.28 (av.0.22) in the case of *Sahouri.*

**Fig. 3:**
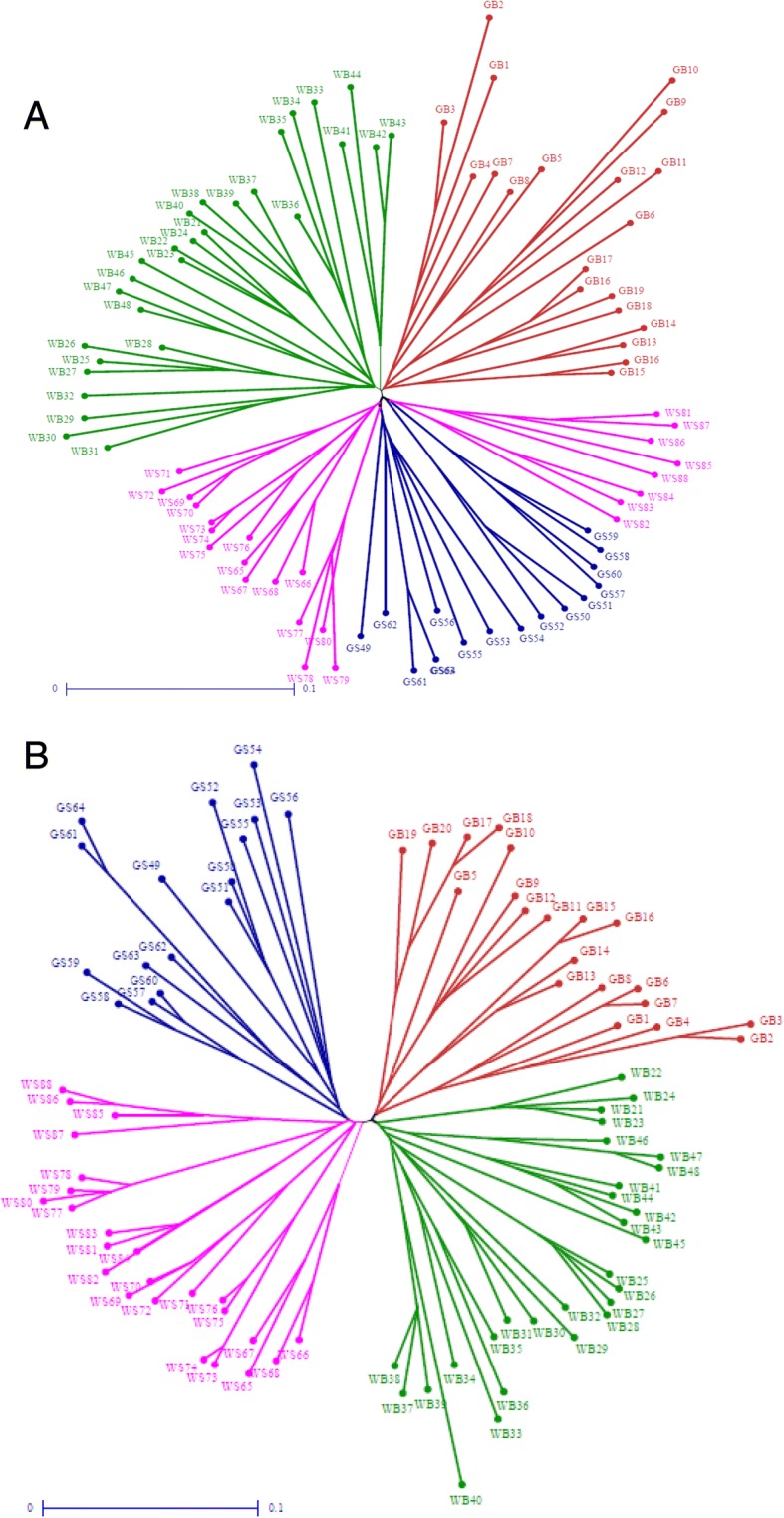
Neighbour-joining analysis of 88 snake melon accessions based on (**a**) DArTseq and (**b**) SNP markers

In order to attain a full picture of the genetic relationships linking the Palestinian snake melon accessions evaluated in this study, NJ trees were constructed using the 7400 DArTseq and 9750 SNP markers (Fig. 3). The tree comprises at least four branches; the results are in agreement with the PCoA and STRUCTURE analyses. Two branches encompass the accessions cultivated in Ramallah, Hebron, and Bethlehem from White Sahouri and Green Sahouri landraces, another branch includes the Green Baladi accessions from Qalqilia, Nablus and Tulkarm; while the last branch included the White Baladi accessions. Grouping of snake melon accessions within the main clusters were in agreement with their folk names and site of collection.

### Population differentiation

Population differentiation was assessed by implementing AMOVA. The percentages of molecular variation among groups (S and B), subgroups (S1, S2, B1, and B2), and individuals are presented in Table 3. Most of the variation (about 90%) was found among individuals, whereas the variations among groups or subgroups were relatively low ranging between 2.12% for groups and 7.91% for subgroups. An apparent differentiation was found between Green Sahouri and Green Baladi while a relatively weak differentiation existed between Green Baladi and White Baladi or Green Baladi and White Sahouri, as could be seen from the distribution of the accessions in the PCoA diagram Fig. 2 both a and b.

**Table 3:** Analysis of molecular variance (AMOVA) based on DArTseq and SNP markers

Source		DArTseq markers		SNP markers	
	Df	Variance Components	Percentage of variation	Variance components	Percentage of variation
Among groups	1	22.866	2.12%	13.934	1.7%
Among subgroups	2	85.303	7.91%	46.031	5.5%
Within individuals	84	969.586	89.96%	773.977	92.8%
Total	87	1077.755	100%	833.942	100%

### Association between DArTseq, SNP markers and geographical location

**Fig. 4:**
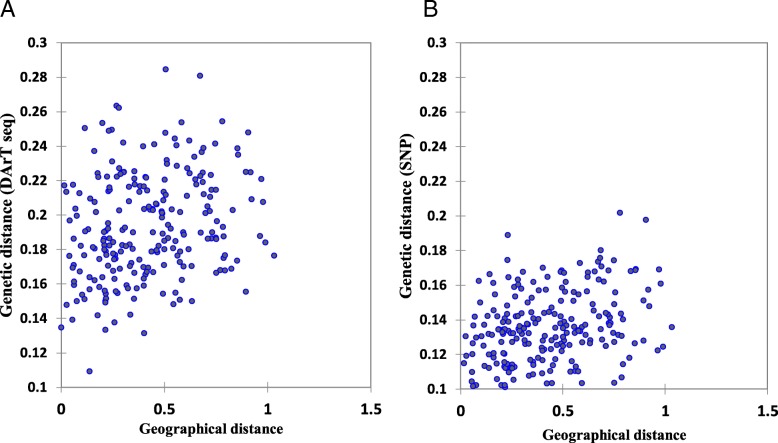
Association between geographical distance and genetic diversity based on (**a**) DArTseq and (**b**) SNP markers

Mantel tests were conducted to determine if there were significant correlations between the matrices of geographic and genetic distances. The results revealed a significant correlation between geographical coordinates and both DArTseq and SNP marker systems (*r* = 0.245, and 0.359, respectively) (Fig. 4).

**Fig. 5:**
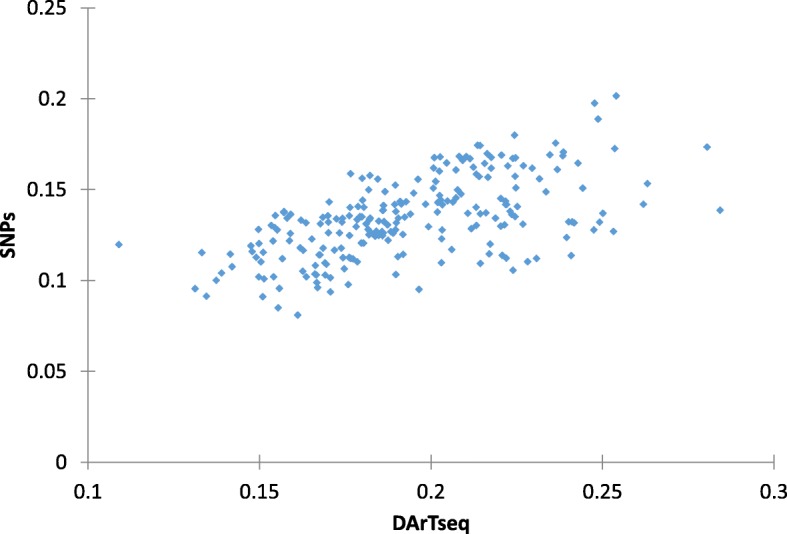
Mantel correlation test between DArTseq and SNP markers

The Mantel test was also carried out to detect correlation between the two genetic diversity systems, comparisons between the clusters generated from DArTseq and SNP marker systems revealed strong association between both systems (*r* = 0.594; *P* < 0.0001) (Fig. 5).

## Discussion

The importance of understanding the genetic diversity of snake melon (*Cucumis melo* var. *flexuosus*) landraces in various geographical regions of Palestine is critical for conservation and utilization of snake melon germplasm, and assisting breeders in developing methods to attain lucrative divergence in the breeding programs [47].

Although several molecular markers have been successfully implemented to investigate the genetic diversity of melons and its close relatives including snake melon [12–15], availability of reliable, robust and economical marker platform is still lacking. In this study, the genetic diversity and population structure analyses of Palestinian snake melon genotypes collected from different areas of the Palestinian West Bank was conducted by GBS technology which offers a rapid, low-cost and high-throughput approach widely utilized to study genetic diversity in cultivated plants [48]. This technology has been a useful approach in recent similar diversity studies, which focused on the use of GBS to distinguish patterns of genetic diversity and genomic features within *Cucumis melo* germplasm and revealed a high genetic variation [43]. To the best of our knowledge, this is the first report on an efficacious application of GBS (DArTseq) for the characterization of snake melon using 9750 SNP and 7400 DArTseq markers which were found to be useful in providing a description of genetic diversity in 88 Palestinian snake melon accessions.

The PIC was used to estimate the informativeness of the DArTseq and SNP markers in this study. Overall, the average PIC of all SNP (PIC = 0.234) and DArTseq markers (PIC = 0.192) were found to be lower than that observed in other crops including durum wheat (PIC = 0.302 and 0.265) for both marker systems, respectively [49]. Approximately 28% and 23% of polymorphic SNP and DArT markers have a PIC value in the range of 0.31–0.50, which, therefore, may be considered informative. These results of the Diversity array technologies (DArTseq and SNP) demonstrate that they could be easily used in this species for more characterization of collections and other genomic studies [27, 50, 51].

Accessions with folk name GS and GB are the most genetically distant landraces, while, the WS and GS accessions have shown to be the most genetically related landraces. This suggests that WS and GS may have evolved from the same source despite the morphological variation between the two landraces. These results are in accordance with our previous research on the morphological characterization of snake melon populations from Palestine where we reported the highest relatedness between the GS and WS accessions [10].

Among the four landraces, weak differentiation was calculated between WS and the two landraces WB and GS (GD = 0.03), and between GB and WB (GD = 0.03), this correlation might be explained by similar morphological characteristics and geographical origin. White Baladi (WB) and WS possess white predominant fruit skin and flesh color, while WS and GS share pale green secondary fruit skin color with stripped pattern, and similar geographical origin. Green Baladi (GB) and WB share the absence of secondary fruit skin color, and similar geographical origin [10].

On the other hand, the lowest similarity indices values were detected between accessions from landraces with folk names GS and GB (GD = 0.05). Accessions of GS were originally collected from the southern region of the West Bank, while GB was collected from the northern region. Moreover, the fruit morphological characters for GS e.g., predominant fruit skin color, secondary fruit skin color, secondary skin color pattern and flesh color are slightly similar to GB landrace. Such information is helpful in terms of the future collection and conservation of Palestinian snake melon landraces and allows us to maintain only a manageable number of samples covering the entire gene pool including the rare alleles [52].

SNP and DArTseq markers used in this study effectively detected the diversity among snake melon accessions. All the three types of DarTseq and SNP diversity analysis (population structure, PCoA, and neighbor-join phylogram) indicated that the different accessions were successfully classified by the marker systems based on geographical origin. Two main groups were identified; all accessions from the *Sahouri* were assigned to S group distributed in the southern Palestinian West Bank (av. similarity index =0.78), while all the *Baladi* accessions were assigned to B group cultivated mainly in the northern West Bank (av. similarity index =0.76).

Further population structure analysis within each group was applied separately using the Bayesian Model-based clustering algorithm; each of the two main groups was further subdivided into two subgroups; accessions having the same folk name were clustered together (S1, White Sahouri and S2, Green Sahouri in S, and B1, Green Baladi and B2, White Baladi in B), which were inferred from the ΔK values that gave K = 2 to both S and B. indicating that the four clearly distinct subgroups (landraces) define the genetic variation of the snake melon cultivated in Palestine.

Validating the methodology, the phylograms derived from the DArT analyses reflect parental relationships between S1 and S2 subgroups grown in the southern region of the West Bank, as they clearly clustered together. These observations support mixing between landraces GS and WS which lead us to believe that GS and WS landraces would have been probably related to each other due to intercrossing and further selection by farmers. Thus, the molecular resolution between WS and GS is negligible despite their morphological differences [10]. All accessions in B1 sub-cluster (GB) grouped separately from WB accessions in B2 sub-cluster. Thus, the molecular resolution between WB and GB is notable; GB and WB both produce moderate size fruits and show significant differences in morphology [10].

Remarkably, population structure, PCoA, and neighbor-joining clustering analyses indicate that patterns of its genetic diversity also depends on its geographical origin. A significant correlation have also been shown between geographical coordinates and DArTseq (*r* = 0.245) and SNP (*r* = 0.359) based on the Mantel correlation test. This information could be accessibly used to position germplasm conservation activities and breeding.

A structured AMOVA was applied to the DArTseq and SNP datasets. The largest fraction of variation was found within populations (78 and 61%), a smaller variation among populations (20 and 35%) and extremely low variation among districts (2 and 4%), which is expected for an outcrossing crop such as snake melon. The high level of variation within populations can be attributed to the gene flow, which depends mostly on informal seed exchanges between farmers from different geographical locations. Furthermore, a plant species’ mating system (allogamy vs. autogamy) highly influences the partitioning of genetic variation among and within populations of the species [53].

## Conclusions

We report the first a DArTseq and SNP analyses results for snake melon (*Cucumis melo* var. *flexuosus*) landraces from Palestine. A large number of these markers and their comparably high polymorphism content are remarkable tools for thorough genome-wide screening to study genetic diversity. Our analyses results for snake melon with both DArTseq and SNP markers, were congruent which could be due to the genotyping thousands of loci without prior sequence information.

Snake melon population structure analyses identified four sub-groups (White Sahouri, Green Sahouri in the southern West Bank, and White Baladi and Green Baladi in the northern West Bank) indicating that the different accessions were successfully classified by the marker systems based on their geographical sources and largely consistent with their folk landraces classification. The four clearly distinct subgroups define the genetic variation of the snake melon cultivated in Palestine. These Palestinian landraces seem to possess unique genes that might allow enrichment of global snake melon gene pool and improvements in its production world-wide.

This genetic diversity of the snake melon gene pool must be further elucidated to facilitate its classification, proper maintenance, conservation and utilization of such valuable resource. The results illustrated here present an advantageous starting point for future genomics studies in snake melon for traits of interest which might prompt molecular breeding approaches adapted to local needs and be a resource for future studies aiming to link genomic variation with evolutionary and phenotypic traits.

Our next objective is to identify genotypes with promising attributes and to conduct association mapping studies focusing on Fusarium-wilt resistance, yield, biotic and abiotic stresses.

## Additional files

Additional file 1: Collection sites and geographical coordinates of Palestinian snake melon accessions used in this study. (DOCX 32 kb)

Additional file 2: Raw sequence data of the 88 snake melon accessions by using a whole genome profiling service for DArTseq and SNP markers. Amplicons containing SNP were located on the melon genome. (XLSX 2649 kb)

Additional file 3: The proportion of membership of 88 snake melon accessions in each cluster and sub cluster (K = 2) as defined with a model-based clustering method from Pritchard et al. (2000) based on 7400 DArT seq markers and 9750 SNP markers. (DOCX 26 kb)

## Abbreviations

AFLP: amplified fragment length polymorphism
AMOVA: analysis of molecular variance
DArT: diversity arrays technology
DarTseq: sequencing-based diversity array technology
GB: Green Baladi
GBS: Genotyping by sequencing
GD: genetic distance
GS: Green Sahouri
He: expected heterozygosity
I: Shannon’s diversity index
ISSR: inter-simple sequence repeat
K: hypothetical clusters
MCMC: Markov chain Monte Carlo
Na: Number of different alleles
Ne: number of effective alleles
NGS: next generation sequencing
P: polymorphic loci
PCoA: principal coordinate analysis
PIC: polymorphic information content
RAPD: random amplified polymorphic DNA
RFLP: restriction fragment length polymorphisms
SNP: single-nucleotide polymorphism
SSR: simple sequence repeat
WB: White Baladi
WS: White Sahouri
