# Supplementary material for: Characterizing Palestinian snake melon (Cucumis melo var. flexuosus) germplasm diversity and structure using SNP and DArTseq markers
Source: BMC Plant Biol. 2018 Oct 19;18:246. doi: 10.1186/s12870-018-1475-2 (PMC6194588; doi:10.1186/s12870-018-1475-2)
Supplement: Supplementary file 1 — Collection sites and geographical coordinates of Palestinian snake melon accessions used in this study. (DOCX 32 kb) [file 12870_2018_1475_MOESM1_ESM.docx]

**Additional file 1****.** Collection sites and geographical coordinates of Palestinian snake melon accessions used in this study.

| **No** | **BERC Gene Bank Number** | **Folk Name** | **Province** | **Collection site** | **landrace** | **Latitude**  **N** | **Longitude**  **E** | **Altitude**  **(Ft)** |
| --- | --- | --- | --- | --- | --- | --- | --- | --- |
| GB1 | BERC-TA13-1 | Baladi akhdar | Tulkarm | Anabta | GB | 32.31 | 35.11 | 552 |
| GB2 | BERC-TA13-2 | Baladi akhdar | Tulkarm | Anabta | GB | 32.31 | 35.11 | 552 |
| GB3 | BERC-TA13-3 | Baladi akhdar | Tulkarm | Anabta | GB | 32.31 | 35.11 | 552 |
| GB4 | BERC-TA13-4 | Baladi akhdar | Tulkarm | Anabta | GB | 32.31 | 35.11 | 552 |
| GB5 | BERC-QJ22-1 | Baladi akhdar | Qalqilia | Jeat | GB | 32.22 | 35.18 | 1270 |
| GB6 | BERC-QJ22-2 | Baladi akhdar | Qalqilia | Jeat | GB | 32.22 | 35.18 | 1270 |
| GB7 | BERC-QJ22-3 | Baladi akhdar | Qalqilia | Jeat | GB | 32.22 | 35.18 | 1270 |
| GB8 | BERC-QJ22-4 | Baladi akhdar | Qalqilia | Jeat | GB | 32.22 | 35.18 | 1270 |
| GB9 | BERC-QH19-1 | Baladi akhdar | Qalqilia | Hajjah | GB | 32.2 | 35.14 | 1365 |
| GB10 | BERC-QH19-2 | Baladi akhdar | Qalqilia | Hajjah | GB | 32.2 | 35.14 | 1365 |
| GB11 | BERC-QH19-3 | Baladi akhdar | Qalqilia | Hajjah | GB | 32.2 | 35.14 | 1365 |
| GB12 | BERC-QH19-4 | Baladi akhdar | Qalqilia | Hajjah | GB | 32.2 | 35.14 | 1365 |
| GB13 | BERC-QG23-1 | Baladi akhdar | Qalqilia | Gensafoot | GB | 32.18 | 35.13 | 1402 |
| GB14 | BERC-QG23-2 | Baladi akhdar | Qalqilia | Gensafoot | GB | 32.18 | 35.13 | 1402 |
| GB15 | BERC-QG23-3 | Baladi akhdar | Qalqilia | Gensafoot | GB | 32.18 | 35.13 | 1402 |
| GB16 | BERC-QG23-4 | Baladi akhdar | Qalqilia | Gensafoot | GB | 32.18 | 35.13 | 1402 |
| GB17 | BERC-NT25-1 | Baladi akhdar | Nablus | Til | GB | 32.2 | 35.2 | 2048 |
| GB18 | BERC-NT25-2 | Baladi akhdar | Nablus | Til | GB | 32.2 | 35.2 | 2048 |
| GB19 | BERC-NT25-3 | Baladi akhdar | Nablus | Til | GB | 32.2 | 35.2 | 2048 |
| GB20 | BERC-NT25-4 | Baladi akhdar | Nablus | Til | GB | 32.2 | 35.2 | 2048 |
| WB21 | BERC-SD32-1 | Baladi abiadh | Salfit | Dear Baloot | WB | 32.07 | 35.03 | 884 |
| WB22 | BERC-SD32-2 | Baladi abiadh | Salfit | Dear Baloot | WB | 32.07 | 35.03 | 884 |
| WB23 | BERC-SD32-3 | Baladi abiadh | Salfit | Dear Baloot | WB | 32.07 | 35.03 | 884 |
| WB24 | BERC-SD32-4 | Baladi abiadh | Salfit | Dear Baloot | WB | 32.07 | 35.03 | 884 |
| WB25 | BERC-JZ03-1 | Baladi abiadh | Jenin | Zababdeh | WB | 32.38 | 35.33 | 1126 |
| WB26 | BERC-JZ03-2 | Baladi abiadh | Jenin | Zababdeh | WB | 32.38 | 35.33 | 1126 |
| WB27 | BERC-JZ03-3 | Baladi abiadh | Jenin | Zababdeh | WB | 32.38 | 35.33 | 1126 |
| WB28 | BERC-JZ03-4 | Baladi abiadh | Jenin | Zababdeh | WB | 32.38 | 35.33 | 1126 |
| WB29 | BERC-JM06-1 | Baladi abiadh | Jenin | Meslyeh | WB | 32.39 | 35.28 | 1242 |
| WB30 | BERC-JM06-2 | Baladi abiadh | Jenin | Meslyeh | WB | 32.39 | 35.28 | 1242 |
| WB31 | BERC-JM06-3 | Baladi abiadh | Jenin | Meslyeh | WB | 32.39 | 35.28 | 1242 |
| WB32 | BERC-JM06-4 | Baladi abiadh | Jenin | Meslyeh | WB | 32.39 | 35.28 | 1242 |
| WB33 | BERC-JB01-1 | Baladi abiadh | Jenin | Bear al-basha | WB | 32.43 | 35.23 | 1256 |
| WB34 | BERC-JB01-2 | Baladi abiadh | Jenin | Bear al-basha | WB | 32.43 | 35.23 | 1256 |
| WB35 | BERC-JB01-3 | Baladi abiadh | Jenin | Bear al-basha | WB | 32.43 | 35.23 | 1256 |
| WB36 | BERC-JB01-4 | Baladi abiadh | Jenin | Bear al-basha | WB | 32.43 | 35.23 | 1256 |
| WB37 | BERC-JA10-1 | Baladi abiadh | Jenin | Mythaloon | WB | 32.35 | 35.27 | 1224 |
| WB38 | BERC-JA10-2 | Baladi abiadh | Jenin | Mythaloon | WB | 32.35 | 35.27 | 1224 |
| WB39 | BERC-JA10-3 | Baladi abiadh | Jenin | Mythaloon | WB | 32.35 | 35.27 | 1224 |
| WB40 | BERC-JA10-4 | Baladi abiadh | Jenin | Mythaloon | WB | 32.35 | 35.27 | 1224 |
| WB41 | BERC-AZ61-1 | Baladi abiadh | Jericho | Jericho | WB | 31.888 | 35.464 | -775 |
| WB42 | BERC-AZ61-2 | Baladi abiadh | Jericho | Jericho | WB | 31.888 | 35.464 | -775 |
| WB43 | BERC-AZ61-3 | Baladi abiadh | Jericho | Jericho | WB | 31.888 | 35.464 | -775 |
| WB44 | BERC-AZ61-4 | Baladi abiadh | Jericho | Jericho | WB | 31.888 | 35.464 | -775 |
| WB45 | BERC-AB60-1 | Baladi abiadh | Jericho | Jericho | WB | 32.386 | 35.489 | -324 |
| WB46 | BERC-AB60-2 | Baladi abiadh | Jericho | Jericho | WB | 32.386 | 35.489 | -324 |
| WB47 | BERC-AB60-3 | Baladi abiadh | Jericho | Jericho | WB | 32.386 | 35.489 | -324 |
| WB48 | BERC-AB60-4 | Baladi abiadh | Jericho | Jericho | WB | 32.386 | 35.489 | -324 |
| GS49 | BERC-BT54-1 | Sahouri akhdar | Bethlahem | Taqoa'a | GS | 31.64 | 35.2 | 2545 |
| GS50 | BERC-BT54-2 | Sahouri akhdar | Bethlahem | Taqoa'a | GS | 31.64 | 35.2 | 2545 |
| GS51 | BERC-BT54-3 | Sahouri akhdar | Bethlahem | Taqoa'a | GS | 31.64 | 35.2 | 2545 |
| GS52 | BERC-BT54-4 | Sahouri akhdar | Bethlahem | Taqoa'a | GS | 31.64 | 35.2 | 2545 |
| GS53 | BERC-BT53-1 | Sahouri akhdar | Bethlahem | Taqoa'a | GS | 31.64 | 35.21 | 2508 |
| GS54 | BERC-BT53-2 | Sahouri akhdar | Bethlahem | Taqoa'a | GS | 31.7 | 35.23 | 1918 |
| GS55 | BERC-BT53-3 | Sahouri akhdar | Bethlahem | Taqoa'a | GS | 31.7 | 35.23 | 1918 |
| GS56 | BERC-BT53-4 | Sahouri akhdar | Bethlahem | Taqoa'a | GS | 31.7 | 35.23 | 1918 |
| GS57 | BERC-BB56-1 | Sahouri akhdar | Bethlahem | Beit Sahour | GS | 31.7 | 35.23 | 1918 |
| GS58 | BERC-BB56-2 | Sahouri akhdar | Bethlahem | Beit Sahour | GS | 31.7 | 35.23 | 1918 |
| GS59 | BERC-BB56-3 | Sahouri akhdar | Bethlahem | Beit Sahour | GS | 31.7 | 35.23 | 1918 |
| GS60 | BERC-BB56-4 | Sahouri akhdar | Bethlahem | Beit Sahour | GS | 31.7 | 35.23 | 1918 |
| GS61 | BERC-BB55-1 | Sahouri akhdar | Bethlahem | Beit Sahour | GS | 31.71 | 35.25 | 1771 |
| GS62 | BERC-BB55-2 | Sahouri akhdar | Bethlahem | Beit Sahour | GS | 31.71 | 35.25 | 1771 |
| GS63 | BERC-BB55-3 | Sahouri akhdar | Bethlahem | Beit Sahour | GS | 31.71 | 35.25 | 1771 |
| GS64 | BERC-BB55-4 | Sahouri akhdar | Bethlahem | Beit Sahour | GS | 31.71 | 35.25 | 1771 |
| WS65 | BERC-RT34-1 | Sahouri abiadh | Ramallah | Trmosayah | WS | 32.04 | 35.29 | 2202 |
| WS66 | BERC-RT34-2 | Sahouri abiadh | Ramallah | Trmosayah | WS | 32.04 | 35.29 | 2202 |
| WS67 | BERC-RT34-3 | Sahouri abiadh | Ramallah | Trmosayah | WS | 32.04 | 35.29 | 2202 |
| WS68 | BERC-RT34-4 | Sahouri abiadh | Ramallah | Trmosayah | WS | 32.04 | 35.29 | 2202 |
| WS69 | BERC-RB38-1 | Sahouri abiadh | Ramallah | Batonia | WS | 31.9 | 35.18 | 2649 |
| WS70 | BERC-RB38-2 | Sahouri abiadh | Ramallah | Batonia | WS | 31.9 | 35.18 | 2649 |
| WS71 | BERC-RB38-3 | Sahouri abiadh | Ramallah | Batonia | WS | 31.9 | 35.18 | 2649 |
| WS72 | BERC-RB38-4 | Sahouri abiadh | Ramallah | Batonia | WS | 31.9 | 35.18 | 2649 |
| WS73 | BERC-RA41-1 | Sahouri abiadh | Ramallah | Dear Ammar | WS | 31.96 | 35.09 | 1773 |
| WS74 | BERC-RA41-2 | Sahouri abiadh | Ramallah | Dear Ammar | WS | 31.96 | 35.09 | 1773 |
| WS75 | BERC-RA41-3 | Sahouri abiadh | Ramallah | Dear Ammar | WS | 31.96 | 35.09 | 1773 |
| WS76 | BERC-RA41-4 | Sahouri abiadh | Ramallah | Dear Ammar | WS | 31.96 | 35.09 | 1773 |
| WS77 | BERC-HS43-1 | Sahouri abiadh | Hebron | Suba | WS | 31.543 | 34.992 | 1568 |
| WS78 | BERC-HS43-2 | Sahouri abiadh | Hebron | Suba | WS | 31.543 | 34.992 | 1568 |
| WS79 | BERC-HS43-3 | Sahouri abiadh | Hebron | Suba | WS | 31.543 | 34.992 | 1568 |
| WS80 | BERC-HS43-4 | Sahouri abiadh | Hebron | Suba | WS | 31.543 | 34.992 | 1568 |
| WS81 | BERC-HH50-1 | Sahouri abiadh | Hebron | Halhol | WS | 31.59 | 35.08 | 2561 |
| WS82 | BERC-HH50-2 | Sahouri abiadh | Hebron | Halhol | WS | 31.59 | 35.08 | 2561 |
| WS83 | BERC-HH50-3 | Sahouri abiadh | Hebron | Halhol | WS | 31.59 | 35.08 | 2561 |
| WS84 | BERC-HH50-4 | Sahouri abiadh | Hebron | Halhol | WS | 31.59 | 35.08 | 2561 |
| WS85 | BERC-HD52-1 | Sahouri abiadh | Hebron | Dora | WS | 31.46 | 35.03 | 2136 |
| WS86 | BERC-HD52-2 | Sahouri abiadh | Hebron | Dora | WS | 31.46 | 35.03 | 2136 |
| W87 | BERC-HD52-3 | Sahouri abiadh | Hebron | Dora | WS | 31.46 | 35.03 | 2136 |
| WS88 | BERC-HD52-4 | Sahouri abiadh | Hebron | Dora | WS | 31.46 | 35.03 | 2136 |
