# Supplementary material for: Characterizing Palestinian snake melon (Cucumis melo var. flexuosus) germplasm diversity and structure using SNP and DArTseq markers
Source: BMC Plant Biol. 2018 Oct 19;18:246. doi: 10.1186/s12870-018-1475-2 (PMC6194588; doi:10.1186/s12870-018-1475-2)
Supplement: Supplementary file 3 — The proportion of membership of 88 snake melon accessions in each cluster and sub cluster (K = 2) as defined with a model-based clustering method from Pritchard et al. (2000) based on 7400 DArT seq markers and 9750 SNP markers. (DOCX 26 kb) [file 12870_2018_1475_MOESM3_ESM.docx]

**Additional file 3.** The proportion of membership of 88 snake melon accessions in each cluster and sub cluster (K = 2) as defined with a model-based clustering method from Pritchard et al. (2000) based on 7400 DArT seq markers and 9750 SNP markers.

| **No** | **Province** | **landrace** | **Cluster /Sub cluster** | **Proportion of membership (Q) to cluster/ sub cluster** | |
| --- | --- | --- | --- | --- | --- |
|  |  |  |  | **DaRT seq markers** | **SNP markers** |
| GB1 | Tulkarm | GB | B/B1 | 0.965/0.98 | 0.916/0.997 |
| GB2 | Tulkarm | GB | B/B1 | 0.991/0.973 | 0.76/0.996 |
| GB3 | Tulkarm | GB | B/B1 | 0.999/0.966 | 0.77/0.998 |
| GB4 | Tulkarm | GB | B/B1 | 0.996/0.981 | 0.959/0.993 |
| GB5 | Qalqilia | GB | B/B1 | 0.887/0.968 | 0.988/0.991 |
| GB6 | Qalqilia | GB | B/B1 | 0.782/0.967 | 0.937/0.992 |
| GB7 | Qalqilia | GB | B/B1 | 0.744/0.983 | 0.948/0.989 |
| GB8 | Qalqilia | GB | B/B1 | 0.658/0.913 | 0.979/0.777 |
| GB9 | Qalqilia | GB | B/B1 | 0.998/0.994 | 0.979/0.936 |
| GB10 | Qalqilia | GB | B/B1 | 0.998/0.991 | 0.975/0.948 |
| GB11 | Qalqilia | GB | B/B1 | 0.996/0.984 | 0.974/0.888 |
| GB12 | Qalqilia | GB | B/B1 | 0.996/0.987 | 0.991/0.934 |
| GB13 | Qalqilia | GB | B/B1 | 0.795/0.985 | 0.748/0.997 |
| GB14 | Qalqilia | GB | B/B1 | 0.635/0.964 | 0.762/0.992 |
| GB15 | Qalqilia | GB | B/B1 | 0.902/0.962 | 0.989/0.886 |
| GB16 | Qalqilia | GB | B/B1 | 0.878/0.932 | 0.995/0.83 |
| GB17 | Nablus | GB | B/B1 | 0.839/0.979 | 0.977/0.998 |
| GB18 | Nablus | GB | B/B1 | 0.941/0.989 | 0.992/0.993 |
| GB19 | Nablus | GB | B/B1 | 0.769/0.98 | 0.843/0.996 |
| GB20 | Nablus | GB | B/B1 | 0.623/0.979 | 0.873/0.998 |
| WB21 | Salfit | WB | B/B2 | 0.974/0.829 | 0.93/0.657 |
| WB22 | Salfit | WB | B/B2 | 0.832/0.795 | 0.94/0.743 |
| WB23 | Salfit | WB | B/B2 | 0.766/0.849 | 0.877/0.681 |
| WB24 | Salfit | WB | B/B2 | 0.945/0.776 | 0.939/0.752 |
| WB25 | Jenin | WB | B/B2 | 0.992/0.989 | 0.982/0.995 |
| WB26 | Jenin | WB | B/B2 | 0.98/0.986 | 0.995/0.998 |
| WB27 | Jenin | WB | B/B2 | 0.996/0.996 | 0.996/0.992 |
| WB28 | Jenin | WB | B/B2 | 0.997/0.989 | 0.995/0.999 |
| WB29 | Jenin | WB | B/B2 | 0.998/0.558 | 0.982/0.82 |
| WB30 | Jenin | WB | B/B2 | 0.931/0.914 | 0.995/0.986 |
| WB31 | Jenin | WB | B/B2 | 0.992/0.807 | 0.97/0.98 |
| WB32 | Jenin | WB | B/B2 | 0.955/0.78 | 0.871/0.768 |
| WB33 | Jenin | WB | B/B2 | 0.997/0.933 | 0.811/0.712 |
| WB34 | Jenin | WB | B/B2 | 0.999/0.953 | 0.985/0.994 |
| WB35 | Jenin | WB | B/B2 | 0.991/0.858 | 0.998/0.922 |
| WB36 | Jenin | WB | B/B2 | 0.971/0.798 | 0.993/0.96 |
| WB37 | Jenin | WB | B/B2 | 0.997/0.96 | 0.922/0.785 |
| WB38 | Jenin | WB | B/B2 | 0.986/0.973 | 0.989/0.995 |
| WB39 | Jenin | WB | B/B2 | 0.998/0.987 | 0.995/0.995 |
| WB40 | Jenin | WB | B/B2 | 0.995/0.981 | 0.992/0.989 |
| WB41 | Jericho | WB | B/B2 | 0.995/0.95 | 0.982/0.877 |
| WB42 | Jericho | WB | B/B2 | 0.867/0.928 | 0.946/0.642 |
| WB43 | Jericho | WB | B/B2 | 0.985/0.952 | 0.959/0.716 |
| WB44 | Jericho | WB | B/B2 | 0.964/0.789 | 0.963/0.914 |
| WB45 | Jericho | WB | B/B2 | 0.682/0.978 | 0.928/0.959 |
| WB46 | Jericho | WB | B/B2 | 0.714/0.973 | 0.94/0.959 |
| WB47 | Jericho | WB | B/B2 | 0.672/0.987 | 0.986/0.989 |
| WB48 | Jericho | WB | B/B2 | 0.606/0.891 | 0.731/0.722 |
| GS49 | Bethlahem | GS | S/S1 | 0.63/0.662 | 0.571/0.818 |
| GS50 | Bethlahem | GS | S/S1 | 0.794/0.959 | 0.512/0.761 |
| GS51 | Bethlahem | GS | S/S1 | 0.836/0.846 | 0.491/0.838 |
| GS52 | Bethlahem | GS | S/S1 | 0.862/0.976 | 0.623/0.674 |
| GS53 | Bethlahem | GS | S/S1 | 0.532/0.995 | 0.731/0.722 |
| GS54 | Bethlahem | GS | S/S1 | 0.87/0.989 | 0.989/0.995 |
| GS55 | Bethlahem | GS | S/S1 | 0.838/0.997 | 0.986/0.997 |
| GS56 | Bethlahem | GS | S/S1 | 0.591/0.904 | 0.99/0.985 |
| GS57 | Bethlahem | GS | S/S1 | 0.617/0.98 | 0.911/0.991 |
| GS58 | Bethlahem | GS | S/S1 | 0.599/0.971 | 0.817/0.95 |
| GS59 | Bethlahem | GS | S/S1 | 0.644/0.943 | 0.87/0.91 |
| GS60 | Bethlahem | GS | S/S1 | 0.57/0.976 | 0.845/0.935 |
| GS61 | Bethlahem | GS | S/S1 | 0.971/0.977 | 0.801/0.97 |
| GS62 | Bethlahem | GS | S/S1 | 0.916/0.995 | 0.884/0.994 |
| GS63 | Bethlahem | GS | S/S1 | 0.996/0.973 | 0.999/0.993 |
| GS64 | Bethlahem | GS | S/S1 | 0.882/0.963 | 0.983/0.987 |
| WS65 | *Ramallah* | *WS* | *S/S2* | *0.63/0.662* | *0.571/0.818* |
| WS66 | Ramallah | WS | S/S2 | 0.794/0.959 | 0.512/0.761 |
| WS67 | Ramallah | WS | S/S2 | 0.836/0.846 | 0.491/0.838 |
| WS68 | Ramallah | WS | S/S2 | 0.862/0.976 | 0.623/0.674 |
| WS69 | Ramallah | WS | S/S2 | 0.821/0.826 | 0.925/0.935 |
| WS70 | Ramallah | WS | S/S2 | 0.967/0.948 | 0.693/0.837 |
| WS71 | Ramallah | WS | S/S2 | 0.998/0.994 | 0.961/0.923 |
| WS72 | Ramallah | WS | S/S2 | 0.998/0.994 | 0.983/0.88 |
| WS73 | Ramallah | WS | S/S2 | 0.995/0.997 | 0.767/0.998 |
| WS74 | Ramallah | WS | S/S2 | 0.97/0.96 | 0.784/0.997 |
| WS75 | Ramallah | WS | S/S2 | 0.998/0.987 | 0.984/0.991 |
| WS76 | Ramallah | WS | S/S2 | 0.998/0.995 | 0.992/0.991 |
| WS77 | Hebron | WS | S/S2 | 0.821/0.853 | 0.998/0.999 |
| WS78 | Hebron | WS | S/S2 | 0.968/0.993 | 0.998/0.997 |
| WS79 | Hebron | WS | S/S2 | 0.993/0.993 | 0.998/0.987 |
| WS80 | Hebron | WS | S/S2 | 0.997/0.997 | 0.998/0.989 |
| WS81 | Hebron | WS | S/S2 | 0.902/0.937 | 0.957/0.992 |
| WS82 | Hebron | WS | S/S2 | 0.917/0.92 | 0.978/0.997 |
| WS83 | Hebron | WS | S/S2 | 0.801/0.903 | 0.824/0.8 |
| WS84 | Hebron | WS | S/S2 | 0.994/0.989 | 0.988/0.987 |
| WS85 | Hebron | WS | S/S2 | 0.809/0.903 | 0.97/0.93 |
| WS86 | Hebron | WS | S/S2 | 0.995/0.997 | 0.832/0.859 |
| WS87 | Hebron | WS | S/S2 | 0.987/0.997 | 0.942/0.939 |
| WS88 | Hebron | WS | S/S2 | 0.848/0.99 | 0.819/0.976 |
